# Growth patterns among HIV-exposed infants receiving nevirapine prophylaxis in Pune, India

**DOI:** 10.1186/1471-2334-12-282

**Published:** 2012-10-31

**Authors:** Malathi Ram, Nikhil Gupte, Uma Nayak, Aarti A Kinikar, Mangesh Khandave, Anita V Shankar, Jayagowri Sastry, Robert C Bollinger, Amita Gupta

**Affiliations:** 1 Johns Hopkins Bloomberg School of Public Health, Dept. of International Health/GDEC, Suite W5506, 615 N. Wolfe Street, Baltimore, MD 21205, USA; 2 BJMC-JHU Clinical Trials Unit, Pune, India; 3 BJ Medical College & Sassoon General Hospitals, Pune, India; 4 Johns Hopkins University School of Medicine, Infectious Diseases, Baltimore, MD, USA; 5 University of Virginia School of Medicine, Department of Health Sciences, Charlottesville, VA, USA; 6 Shrimati Kashibai Navale Medical College & Hospital, Narhe Pune, India

**Keywords:** HIV-exposed infants, Growth patterns, India, Extended use of nevirapine, Risk factors, Timing of HIV Infection

## Abstract

**Background:**

India has among the highest rates of infant malnutrition. Few studies investigating the growth patterns of HIV-exposed infants in India or the impact of timing of HIV infection on growth in settings such as India exist.

**Methods:**

We used data from the Six Week Extended Nevirapine (SWEN) trial to compare the growth patterns of HIV-infected and HIV-exposed but uninfected infants accounting for timing of HIV infection, and to identify risk factors for stunting, underweight and wasting. Growth and timing of HIV infection were assessed at weeks 1, 2, 4, 6, 10, 14 weeks and 6, 9, 12 months of life. Random effects multivariable logistic regression method was used to assess factors associated with stunting, underweight and wasting.

**Results:**

Among 737 HIV-exposed infants, 93 (13%) were HIV-infected by 12 months of age. Among HIV-infected and uninfected infants, baseline prevalence of stunting (48% vs. 46%), underweight (27% vs. 26%) and wasting (7% vs. 11%) was similar (p>0.29), but by 12 months stunting and underweight, but not wasting, were significantly higher in HIV-infected infants (80% vs. 56%, 52% vs. 29%, p< 0.0001; 5% vs. 6%, p=0.65, respectively). These differences rapidly manifested within 4–6 weeks of birth. Infants infected in utero had the worst growth outcomes during the follow-up period. SWEN was associated with non-significant reductions in stunting and underweight among HIV-infected infants and significantly less wasting in HIV-uninfected infants. In multivariate analysis, maternal CD4 < 250, infant HIV status, less breastfeeding, low birth weight, non-vaginal delivery, and infant gestational age were significant risk factors for underweight and stunting.

**Conclusion:**

Baseline stunting and underweight was high in both HIV-infected and uninfected infants; growth indices diverged early and were impacted by timing of infection and SWEN prophylaxis. Early growth monitoring of all HIV-exposed infants is an important low-cost strategy for improving health and survival outcomes of these infants.

**Trial Registration:**

NCT00061321

## Background

Poor growth, as a result of inadequate nutritional intake and/or increased susceptibility to infections, poses an increased risk of mortality among children [1]. Data from 53 developing countries found that the percentage of child deaths attributable to the potentiating effects of malnutrition ranged from 13% to 67%, and that 83% of malnutrition-related deaths were related to mild-to-moderate malnutrition (weight-for-age <80% of median) rather than severe malnutrition (weight-for-age <60% of median), highlighting the significant impact of nutritional status on child survival [2-5].

HIV-infection further negatively impacts growth and manifests as weight loss as well as compromised ponderal and linear growth [6,7]. Among infants exposed to HIV- infection in utero, during birth, or postnatally through breastfeeding, growth faltering or failure is now recognized as early markers of HIV infection, disease progression and as a prognostic tool for survival [7,8]. Abnormal growth patterns in HIV-infected children have been documented in both developed and developing country settings [9-20]. For children that are already suffering with poor nutritional status, concurrent HIV infection poses substantial additional risks for morbidity and mortality.

Despite the significant literature on infant growth and HIV in resource-constrained settings, a recent review noted that the vast majority of research comes from countries of Africa and that very few studies have incorporated the data of timing of transmission (assessed by repeated PCR measures) to assess differences in growth [9]. India has the world’s third largest number of HIV-infected individuals, nearly half of the pediatric population lives in poverty and malnutrition of infants and young children is a significant public health problem (38% of those under 3 years of age are stunted, 19% wasted, and 46% underweight) [21]; these proportions are higher than in most countries in Africa. To date, there have been no studies in India comparing growth patterns of HIV-infected infants with those of HIV-exposed but uninfected infants. Furthermore, the factors associated with poor growth among HIV-exposed infants in India could be very different from those evidenced in African countries given the higher prevalence of malnutrition in poor Indian children in general.

The objectives of our study were to: a) compare the growth patterns of HIV-infected and HIV-exposed but uninfected infants and account for timing of HIV infection, and b) identify risk factors for stunting, underweight and wasting. We used data from the Six Week Extended Nevirapine (SWEN) prevention of mother to child HIV transmission trial, India’s only Phase III trial to examine HIV prevention in breastfed infants, to address our objectives. We also assessed the impact of the new WHO recommended strategy of extended nevirapine prophylaxis given to breastfed infants on growth indices as previously we had noted a mortality benefit among children exposed to extended nevirapine and we hypothesized that this benefit may be manifested by improved growth patterns independent of HIV status.

## Methods

### Population and study design

The SWEN study enrolled HIV-infected pregnant women who were attending the antenatal clinic and/or delivery ward of Sassoon General Hospital, the urban public hospital of Byramji Jeejeebhoy Medical College (BJMC) located in Pune, Maharashtra, India between 2002 and 2007. The details of this trial have been published elsewhere [22]. HIV-infected women who indicated an intention to breastfeed their infants and provided informed consent were eligible for study enrollment. Written consent was obtained where possible; for eligible women who could not read, consent was obtained orally and documented in writing by a witness. The criteria for enrollment included: intention to breastfeed their infant, ≥18 years of age, gestational age ≥24 weeks, hemoglobin > 7.5 gm/dl, creatinine <1.2 mg/dl, liver function tests < 3 times the upper limit of normal, and lack of serious pregnancy complications. Live-born infants were randomized to receive either a single dose nevirapine (SdNVP) within 72 hours of birth or an extended nevirapine prophylaxis (SWEN) during the first six weeks of life if they met the following criteria within 7 days after birth: hemoglobin > 7.5 gm/dl, SGPT < 5 times upper limit of normal values, and serum creatinine <1.0 mg/dl. All infants were also given daily multivitamins (VI-SYNERAL) from day 8 to 42 days after birth. In case of twin birth, both the siblings were enrolled in the same cohort and received the same random treatment assignment.

### Follow-up procedures

Mother-infant pairs were followed prospectively for up to 12 months postpartum with eleven scheduled visits occurring at 1, 2, 3, 4, 5, 6, 10, 14 weeks and at 6, 9 and 12 months. At each visit, except weeks 3 and 5, anthropometric measurements, clinical assessments and laboratory investigations were completed. Determination of qualitative HIV infection was done with an in-house, externally validated HIV DNA PCR assay developed at the National AIDS Research Institute in Pune, India. A positive DNA PCR test was confirmed by a quantitative HIV-1 RNA PCR of >5000 copies/ml during the next follow-up visit using Roche Amplicor HIV-1 Monitor test, version 1.5 (F Hoffmann-La Roche Ltd, Basel, Switzerland), and was externally quality assured as described elsewhere [22]. An infant was determined to be infected with HIV if two independent HIV PCR tests were positive at different time points or if one test was HIV PCR positive and there were no subsequent infant samples available for testing.

### Anthropometric measurements

Birth weight and length were obtained immediately after birth in the delivery room or postpartum ward, as appropriate. Subsequent anthropometric measurements were performed by an experienced study nurse trained in standard anthropometric techniques. Weight was measured to the nearest 100 grams using a standard weighing scale, and length was measured to the nearest 0.1 cm in a recumbent position using an infantometer. Measurements were taken in duplicate and if more than 10% discrepant, a third measurement was taken.

The SWEN trial was approved by the Johns Hopkins and Pune institutional review boards, and BJMC Ethics Committee.

### Statistics

Standardized Z-scores for Weight-for-Age (WAZ, referring to underweight), Length-for-Age (LAZ, referring to stunting) and Weight-for-Length (WLZ, referring to wasting) were calculated using the WHO Anthro version 2.0 software [23]. The Z-score measures the number of standard deviations above/below the median for age and gender of a reference population, drawn from The WHO Multicentre Growth Reference Study, an internationally applicable standard that includes children from a diverse set of countries: Brazil, Ghana, India, Norway, Oman and the USA [24]. We defined poor growth as categorical variables using the following three anthropometric indices: *Underweight*, if WAZ score was <−2.0 SD units; *Stunted*, if LAZ was <−2.0 SD units; and *Wasted*, if WLZ was <−2.0 SD units. Since infants in our study were randomized to receive SWEN or SdNVP, we examined whether the SWEN dose afforded any additional benefit to the infants as evidenced by their anthropometric indices and whether HIV infection had any impact on growth.

Infants were categorized as HIV-infected in two ways: 1) if an infant was found to be HIV positive anytime during the follow-up period of 12 months, or negative otherwise; and 2) depending on timing of infection, he/she was categorized as in utero infected if found to be HIV positive within 48 hours of birth; infected peripartum if found to be HIV positive after 48 hours but before 6 weeks after birth; or infected postpartum if found to be HIV positive after age 6 weeks and older.

For continuous variables, comparison of median values of maternal and infant covariates between the HIV-infected infants and HIV-exposed but uninfected infants was done using Wilcoxon Mann–Whitney *U* test; for categorical variables, the comparison were based on *χ*^2^ test or Fisher’s exact test as appropriate. In order to identify factors associated with underweight, stunting, and wasting, we used random effects logistic regression modeling methodology using several maternal and infant covariates. Maternal variables included in the analyses were: age, religion, marital status, family type, educational status, employment status, parity, gestational age at delivery, hemoglobin levels at delivery, CD4 cell count/mm^3^, and HIV quantitative RNA (i.e.viral load) in log_10_ units. Infant covariates included in the analyses were: gender, time-dependent HIV status, time-dependent not breastfeeding, interaction term between time-dependent HIV status and not breastfeeding, birth weight, treatment arm, prematurity, delivery mode, and hospitalization, as a proxy for severity of illnesses, during follow-up period. For the multivariate analyses, we adjusted for those variables with a p-value of <0.05 in the univariate analyses. Variables for treatment arm, HIV status and whether or not being breastfed were forced into the model even if the p-value was not significant in the univariate analysis (p > 0.05) since they were primary variables of interest for this paper. Statistical analyses were performed using STATA [25] Version 10 software for personal computers, and in all cases the level of significance was established at <0.05.

## Results

Among 737 live-born infants born to 730 HIV-infected mother, 93 (12.6%) were HIV-infected infants (*Infected cohort*) and 644 were HIV-exposed but uninfected infants (*Uninfected cohort*) by 12 months of life. There were 7 set of twins in the study sample. Of 737 infants, 28 (3.8%) infants were in utero HIV-infected, 10 (1.4%) were peripartum infected (within 6 weeks after birth) and 55 (7.5%) were postpartum infected (after 6 weeks of birth). Table 1 shows the comparison of baseline maternal and infant characteristics between the two cohorts. Mothers of *infected* infants had significantly lower median hemoglobin levels (10.1 vs. 10.8, p<0.0001), lower median CD4 cell counts (320 vs. 470, p<0.0001) and higher log_10_ viral load (4.7 vs. 3.8, p<0.0001) compared to those in the *uninfected* cohort. Both cohorts had a similar median duration of breastfeeding (98 days vs. 98 days) and proportion breastfed for 1–4 months (81% vs. 79%). A significantly higher proportion of infants in the *infected* cohort were hospitalized for illnesses during the follow-up period compared to those in the *uninfected* infants (37% vs. 18%, p<0.0001). Only 16 HIV-infected infants were on HAART during the follow-up period.

**Table 1:** Maternal and infant characteristics by exposure to infant HIV status (N=737)

**Characteristics**	**Total N = 737**	**HIV -uninfected N = 644**	HIV- infected* N = 93	**P-value**^#^
***Baseline Maternal Characteristics***				
Age in years, Median (IQR)	23 (21,25)	23 (21,25)	24 (21,26)	0.082
Hindu Religion, n (%)	570 (78)	493 (77)	77 (83)	0.212
Married, n (%)	714 (97)	624 (98)	90 (97)	0.681
Nuclear Family, n (%)	351 (48)	306 (48)	45 (48)	0.928
≤ Primary School Education, n (%)	294 (40)	250 (39)	44 (47)	0.129
Housewife/Unemployed, n (%)	594 (81)	525 (82)	69 (74)	0.066
Parity, Median (IQR)	1 (0, 2)	1 (0, 2)	1 (1, 2)	**0.004**
Gestational Age at Delivery, Median (IQR)	32 (28,35)	32 (28,35)	32 (26,34)	0.069
Hemoglobin at Delivery, Median (IQR)	10.7 (9.2, 11.9)	10.8 (9.2, 10.8)	10.1 (8.8, 11.5)	**<0.0001**
CD_4_ Counts (in 100 s), Median (IQR)	4.6 (3.1, 6.5)	4.7 (3.34, 6.4)	3.2 (1.9, 5.3)	**<0.0001**
log_10_ Viral Load, Median (IQR)	3.9 (3.2, 4.5)	3.8 (3.1, 4.4)	4.7 (4.0, 5.1)	**<0.0001**
***Baseline Infant Characteristics***				
Male Gender, n(%)	390 (53)	343 (53)	47 (51)	0.623
HIV Positive at Birth, n (%)	28 (4)	--	28 (30)	--
Birth Weight, Median (IQR)	2.6 (2.4, 3.0)	2.7 (2.4, 3.0)	2.6 (2.5, 2.6)	0.292
Low Birth Weight (< 2.5 kg) n (%)	207 (28)	181 (28)	26 (28)	0.941
Birth Length, Median (IQR)	46 (45, 48)	46 (45, 48)	46 (45, 47)	0.865
Weight for Height Z-score, Median (IQR)	−0.03 (−1.1, 0.6)	−0.03 (−1.2, 0.7)	−0.04 (−0.8, 0.6)	0.908
Wasting, n (%)	62 (10)	57 (11)	5 (7)	0.299
Length for Age Z-score, Median (IQR)	−1.7 (−2.2, –1.0)	−1.7 (−2.2, –1.0)	−1.7 (−2.6, –1.2)	0.922
Stunting, n (%)	339 (47)	295 (46)	44 (48)	0.795
Weight for Age Z-score, Median (IQR)	−1.5 (−2.0, –0.7)	−1.5 (−2.0, –0.7)	−1.7 (−2.0, –0.7)	0.408
Underweight, n (%)	190 (26)	165 (26)	25 (27)	0.828
Infant Gestational Age in Weeks, Median (IQR)	38 (38, 38)	38 (38, 38)	38 (38, 38)	0.434
Hemoglobin at Birth, Median (IQR)	17.3 (15.7, 18.7)	17.3 (15.7, 18.8)	17.3 (15.7, 18.5)	0.868
Normal Vaginal Delivery, n (%)	590 (80)	513 (80)	77 (83)	0.513
***Follow-up Variables***				
Exclusive Breastfeeding (days), Median (IQR)	98 (97, 100)	98 (97, 100)	98 (72, 100)	0.606
Exclusive Breastfeeding, n (%)				
< 1 Month	61 (8)	54 (8)	7 (8)	0.741
1 – 4 Months	587 (80)	512 (79)	75 (81)	
4 – 6 Months	46 (6)	42 (6)	4 (4)	
>6 Months	43 (6)	36 (6)	7 (8)	
Grade 3 or 4 Adverse Event, n (%)	261 (35)	214 (33)	47 (51)	**0.001**
Hospitalization, n (%)	156 (21)	122 (19)	34 (37)	**<0.0001**

* HIV-infected group includes infants infected at any time during the 12-month follow-up period.

^**#**^ P values for comparison of median values based on Wilcoxon Mann–Whitney *U *test, and for comparison of proportions, based on χ^2 ^test.

### Anthropometric Indices by Infant HIV Status

**Figure 1:**
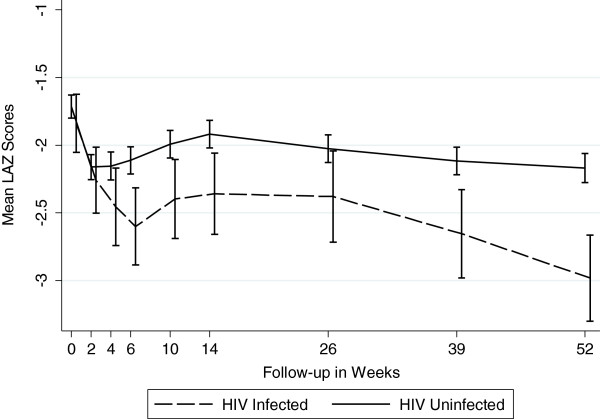
Mean Length -for-Age Z Scores by Infant HIV Status (Stunting).

**Figure 2:**
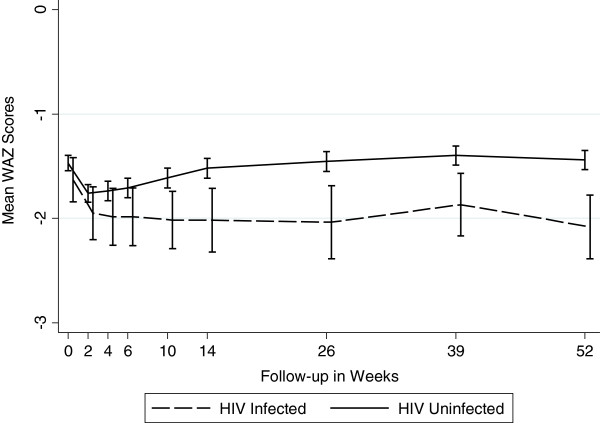
Mean Weight-for-Age Z Scores by Infant HIV Status (Underweight).

**Figure 3:**
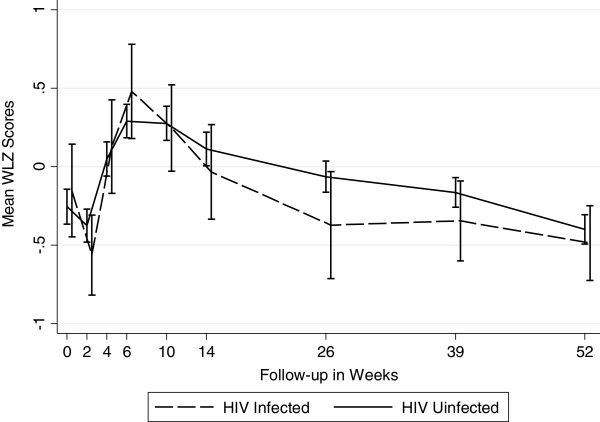
Mean Weight-for-Length Z Scores by Infant HIV Status (Wasting).

Figures 1, 2 and 3 graphically display the differences in the anthropometric indices (LAZ, WAZ and WLZ) at each time point using a dichotomized definition of HIV-infection during 12 month follow-up period (ie HIV-infected or HIV-exposed uninfected). At birth, both the *infected* and the *uninfected* cohort had a similar high proportion of stunting (48% vs. 46%), underweight (27% vs. 26%), and wasting (7% vs. 11%), respectively (p>0.29) [Additional file 1: Tables S1, S2 and S3]. By 12 months, 80% of infants in the *infected* cohort were significantly more stunted compared to 56% in the *uninfected* cohort (p<0.0001). The stunting among the *infected* cohort emerged early and became statistically significant at week 4 and persisted through to 12 months of age; (12 month LAZ *infected* cohort −2.98 vs. *uninfected* cohort −2.17, p<0.0001).

A similar trend was seen for underweight, with infants in the *infected* cohort more underweight (lower WAZ scores) during the follow-up period compared to *uninfected* infants, with statistically significant differences arising at week 6 and persisting through to 12-months of life. At 12 months, 52% of *infected* cohort was underweight compared to 26% in the *uninfected* cohort (WAZ −2.08 vs. –1.44, p<0.0001).

There were no significant differences in the mean wasting (WLZ) between infants in the two cohorts throughout the follow-up period, except at 6 months, when infants in the *infected* cohort had statistically significantly worse WLZ score compared to those in the *uninfected* cohort (−0.37 vs. –0.06, p=0.043). Prevalence of wasting (<−2.0 WLZ score) at 12 months was about 6% among infants in both cohorts.

### Impact of timing of HIV transmission

**Figure 4:**
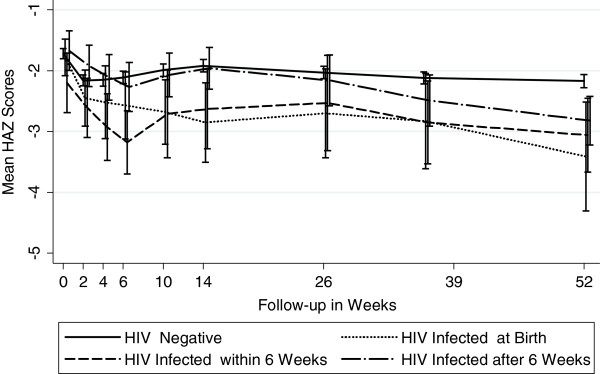
Mean Length -for-Age Z Scores by Timing of HIV Transmission (Stunting).

**Figure 5:**
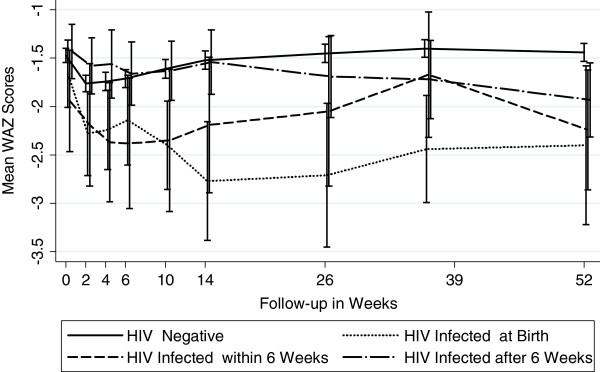
Mean Weight -for-Age Z Scores by Timing of HIV Transmission (Underweight).

**Figure 6:**
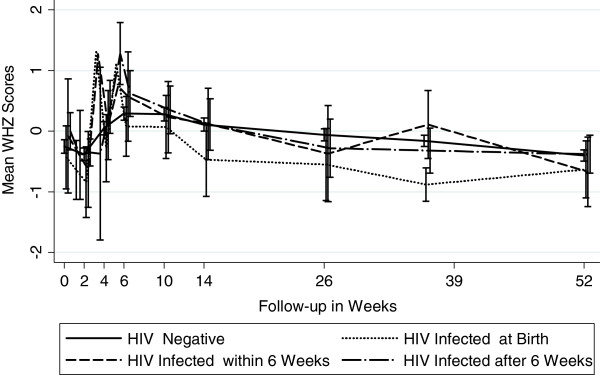
Mean Weight -for-Length Z Scores by Timing of HIV Transmission (Wasting).

Figures 4, 5 and 6 show the anthropometric indices by timing of HIV infection. In utero and peripartum infected infants had similar stunting up to 9 months but by 12 months, those infected in utero had more stunting (worse LAZ scores). Infants postpartum infected after 6 weeks had growth stunting patterns similar to those uninfected upto 6 months after which the LAZ scores diverged dramatically compared to those uninfected. WAZ scores among in utero infected infants were lower throughout follow-up period of 12 months compared to those infected peripartum or postpartum. The growth curves of uninfected infants and those postpartum infected diverged around 14 weeks and continued to remain divergent throughout the subsequent follow-up period. For WLZ scores, infants infected at birth had lower scores from 6 weeks on. The WLZ curves for those infected within 6 weeks, those infected after 6 weeks, and infants uninfected followed a similar pattern.

### Impact of extended nevirapine prophylaxis

**Figure 7:**
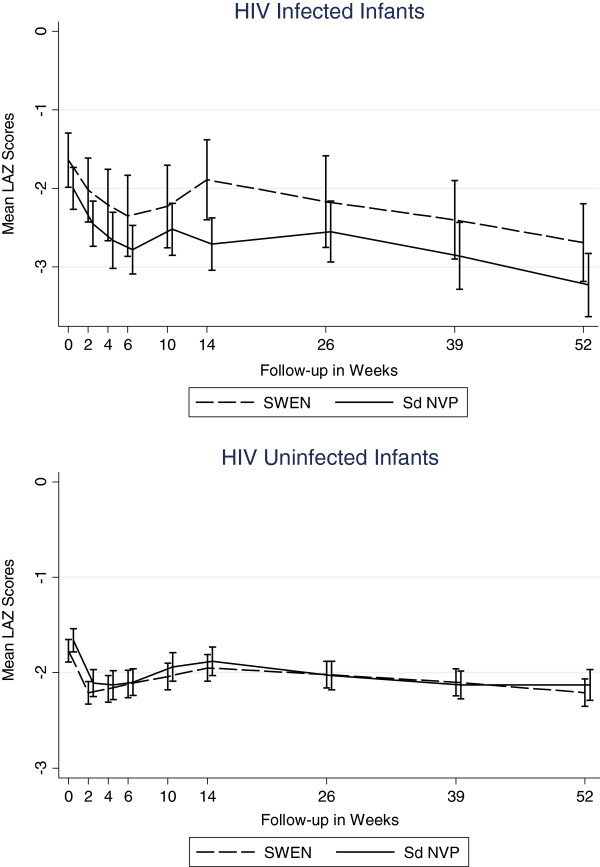
Mean Length-for-Age Z Scores by SWEN Arm and Infant HIV Status (Stunting).

**Figure 8:**
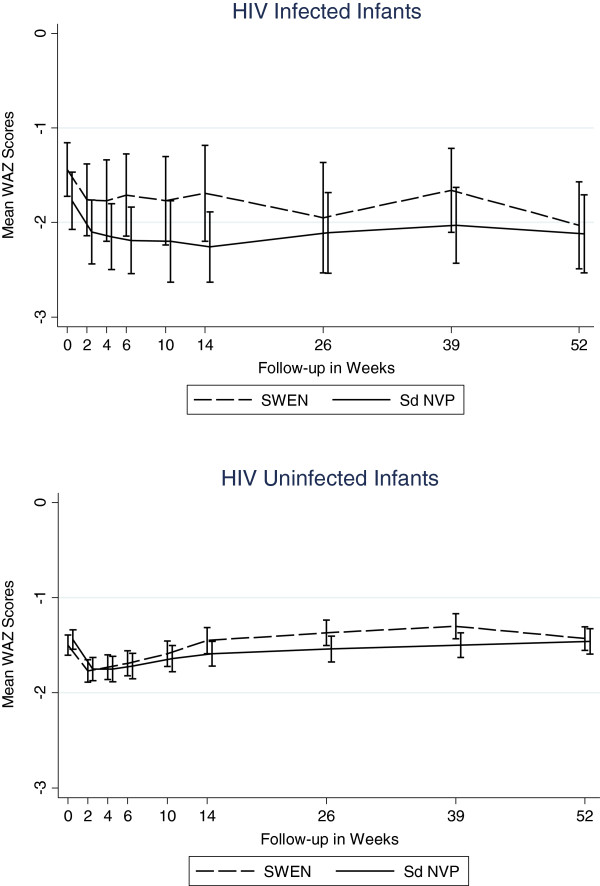
Mean Weight-for-Age Z Scores by SWEN Arm and Infant HIV Status (Underweight).

**Figure 9:**
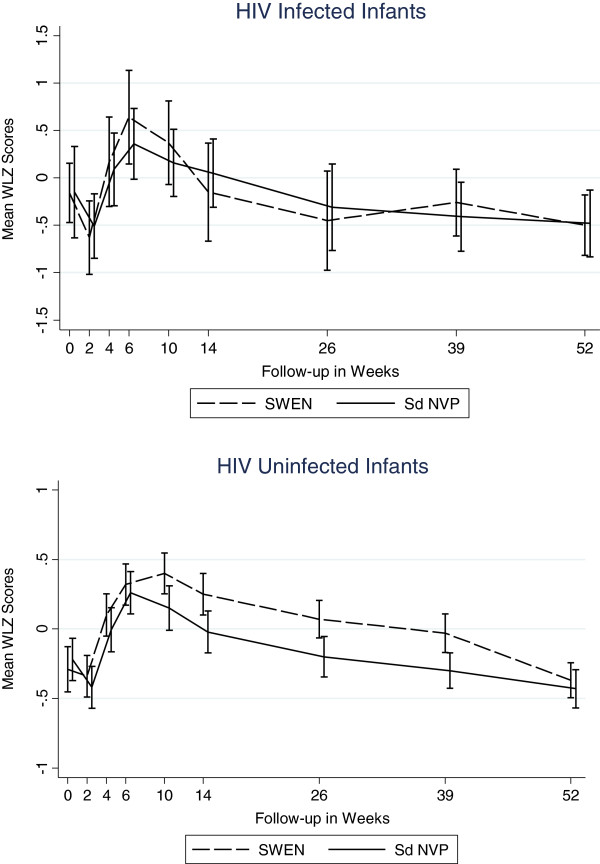
Mean Weight-for-Length Z Scores by SWEN Arm and Infant HIV Status (Wasting).

While SWEN exposure among infants in the *infected* cohort appeared to have a beneficial effect on LAZ and WAZ scores, these differences did not reach statistical significance. There was no clear pattern in the impact of SWEN on wasting (WLZ) in the *infected* cohort (Figures 7, 8, and 9). SWEN however was associated with significantly less wasting among infants in the *uninfected* cohort between week 10 and month 9, a difference in Z score ranging from 0.25 to 0.27, p≤0.024 (Additional file 1: Tables S4, S5 and S6).

### Risk Factors for Poor Growth

In multivariate analyses, several factors were identified as being independently associated with malnutrition (Table 2). Infants whose mothers had primary education or less were more likely to be stunted, but not underweight or wasted, when compared to those with education levels above primary school. Infants born to mothers with CD4 counts below 250 cells/mm^3^ had a higher risk of being stunted and underweight, while a high maternal HIV viral load at delivery was associated with increased the risk of wasting. Infants who were HIV-infected and not breast-fed were at a higher risk of being stunted and underweight, but not wasted, and the interaction term between these two variables was not statistically significant. The factors that had a similar impact on all measures of malnutrition were: birth weight below 2.5 Kg, gestational age below 37 weeks and delivery mode other than normal vaginal delivery -- all three substantially increasing the risk of stunting, underweight and wasting. Maternal anemia significantly increased the risk of stunting but not underweight. Infant morbidity, as measured by whether the infant was hospitalized or not, increased the risk of underweight and wasting, but not stunting.

**Table 2:** Multivariate random-effects logistic regression results

	**Stunting (<−2 SD LAZ)**		**Underweight (<−2 SD WAZ)**		**Wasting (<−2 SD WLZ)**	
	**Odds ratio (CI)**	**P-value**	**Odds ratio (CI)**	**P-value**	**Odds ratio (CI)**	**P-value**
***Maternal Characteristics***						
Less than Primary Education	1.57 (1.11, 2.23)	**0.012**	1.20 (0.84, 1.73)	0.318	--	
Housewife/Unemployed	0.79 (0.51, 1.23)	0.305	0.84 (0.53, 1.31)	0.433	--	
Hemoglobin <9 g/dL	1.76 (1.14, 2.72)	**0.011**	1.31 (0.83, 2.06)	0.240	--	
CD4 <250	2.03 (1.22, 3.38)	**0.006**	1.73 (1.03, 2.91)	**0.038**	--	
log_10_ Viral Load	1.06 (0.86, 1.31)	0.606	1.16 (0.93, 1.46)	0.199	1.24 (1.02, 1.52)	**0.034**
***Infant Characteristics***						
Male Gender	1.84 (1.30, 2.60)	**0.001**	--^#^		--	
SWEN	0.89 (0.63, 1.25)	0.498	0.76 (0.53, 1.08)	0.123	0.71 (0.51, 0.99)	**0.046**
HIV Status (Time Dependent)	2.33 (1.23, 4.43)	**0.010**	2.09 (1.15, 3.82)	**0.016**	1.24 (0.61, 2.52)	0.554
Not Breastfeeding (Time dependent)	1.31 (1.11, 1.55)	**0.002**	0.83 (0.69, 1.00)	**0.047**	0.86 (0.65, 1.14)	0.293
HIV Status * Not Breastfeeding Interaction	0.99 (0.48, 2.04)	0.985	1.83 (0.92, 3.65)	0.087	0.84 (0.33, 2.10)	0.708
Low Birth Weight (< 2.5 kg)	11.00 (6.82, 17.75)	**<0.0001**	41.56 (26.04, 66.34)	**<0.0001**	2.83 (1.87, 4.26)	**<0.0001**
Gestational Age <37 weeks	1.79 (1.37, 5.71)	**0.005**	5.34 (2.69, 10.60)	**<0.0001**	1.93 (1.13, 3.29)	**0.015**
Delivery mode other than normal vaginal	1.99 (1.30, 3.07)	**0.002**	2.63 (1.70, 4.07)	**<0.0001**	1.92 (1.30, 2.84)	**0.001**
Hospitalization	1.24 (0.81, 1.89)	0.323	2.16 (1.40, 3.32)	**0.001**	1.62 (1.10, 2.38)	**0.014**

^# ^Not included in the multivariate model.

We conducted a time dependent analysis on growth z-scores stratified by vital status and HIV status. Among the *infected* cohort, infants who died had a significantly poor growth pattern than those who were alive at 12 months of age. Similar results were observed in the HIV *uninfected* cohort (Data not shown but are available on request). We conducted additional analysis excluding low birth weight infants to examine if preexisting malnutrition had any impact on our results, however, we found that the results of this analysis were similar to the one including all infants and didn't alter our conclusions.

## Discussion

Our data on infant growth among HIV-exposed infants from the SWEN trial in India highlight several important findings. First, we found a high prevalence of baseline malnutrition among both HIV-infected and HIV-exposed, uninfected infants. We observed that differences in stunting and underweight between HIV-infected and uninfected infants emerged early and persisted throughout the first year of life. Secondly, we found that SWEN exposure was associated with lower risk of wasting in HIV-exposed, uninfected infants. Lastly, we confirmed the impact of maternal and infant factors on growth -- low maternal education, maternal advanced disease state as measured by CD4 count and viral load, low infant birth weight, infant HIV infection and morbidity were the key independent factors associated with poor growth outcome. As expected, breastfed infants were found to have better growth outcomes compared to those not breastfed.

The latest national nutritional status data for Indian infants 0–6 months in the general population showed 23% stunting, 32% underweight, and 31% wasting [26]. The nutritional status of infants in the same age group in some of the African countries is comparatively better than those in India, with 9% 19% stunted, 4%-14% underweight, and 3%-16% wasted (Additional file 1: Table S7). Hence, the baseline levels of malnutrition among Indian infants in the community are far worse to begin with than in many other resource-constrained regions.

In our study, there was an overall high prevalence of malnutrition at birth with HIV-exposed infants having approximately 47% stunting, 10% wasting and 26% underweight. In a retrospective study of 162 HIV-exposed infants at a Regional Pediatric Center for HIV in Delhi, prevalence of wasting and stunting was 50.5% and 48.8%, respectively [27]. While our prevalence of stunting was comparable to that in this study, the wasting was lower in our study. In a cohort of antiretroviral-naïve HIV-infected infants in south India, the prevalence of stunting, underweight and wasting was 58%, 63% and 16%, respectively [28]. In contrast to Indian studies, one in Dar es Salaam, Tanzania found less baseline stunting (29%) but similar wasting prevalence (8%) [20]. By 6 months of life, stunting in both HIV-exposed but uninfected (51%) and HIV-infected infants (63%) in our study were significantly worse off compared to infants 0–6 months of age in the general population in India (23%) [26]. In a review of 6 infant growth studies examining HIV exposure and postnatal growth outside of India, only one study in Kenya showed significantly more stunting among HIV-exposed uninfected compared to infants unexposed to HIV. The other studies found a lack of association suggesting that viral exposure without infection is not detrimental to postnatal growth [9]. In our study, HIV-exposed but uninfected infants also had poor growth outcomes, although HIV-infected infants had far worse outcomes. This could partly be explained by the overall high levels of malnutrition at birth in our sample.

Among *infected* cohort in our study, baseline LAZ, WAZ, and WLZ scores were −1.84, –1.63 and −0.15 respectively as compared to the better LAZ, WAZ and WLZ (−0.62, –0.83, and −1.11 respectively), reported among HIV-infected infants in Democratic Republic of Congo [16] or Durban, South Africa [10] (0.13, 0.10, and −0.43) respectively. By 12 months, *infected* cohort in our study were far worse off especially with respect to LAZ and WAZ scores (−2.98 and −2.08 respectively), as compared to infected infants in the Democratic Republic of Congo study (−1.67 and −1.86, respectively), or Durban, South Africa study (−1.26 and −0.53, respectively).

We confirmed the compounding impact HIV infection has on growth as measured by LAZ and WAZ scores. With onset of HIV infection, the divergence in growth profile in our infants occurred within 4–6 weeks of life and persisted through one year of life. Other studies have noted differences occurring around 3 months of age [13,16,17], but most have noted the difference in growth between HIV-infected and uninfected occurring by one year of life [8,9,27]. LAZ score among *infected* infants in our study was lower by 0.35 at 6 months and 0.81 at 12 months. In other studies where negative association was found, HIV-infected infants had lower LAZ scores by 0.23 to 1.55 at 6 months of age, and 0.25 to 0.72 at 12 months [9]. WAZ scores among HIV-infected infants were lower by 0.59 at 6 months and 0.64 at 12 months. The corresponding numbers reported by other studies ranged from 0.20 to 1.72 at 6 months and 0.17 to 0.87 at 12 months. Clearly, infants in our study were worse off both in terms of LAZ and WAZ. Some studies have reported that differences in weight between the two groups were detected around the same time that differences in height [9] were detected, which is consistent with what we found in our study. As in other studies, we found that the differences in WLZ scores between the two groups were inconsistent and were not statistically significant.

With regard to the timing of infection, not surprisingly those infected at birth have worse WAZ and WLZ during the follow-up period compared to those infected within 6 weeks and those infected after 6 weeks. Critical factors in preventing in utero infection and associated poor growth are getting pregnant women into prenatal care, rapidly testing them for HIV and starting those that are HIV-infected with antiretrovirals as soon as possible. Furthermore protecting infants while being breastfed with regimens such as SWEN are important for preventing HIV and poor growth outcomes overall. We found that infants who remained HIV-uninfected and received SWEN were less likely to be wasted, but SWEN did not significantly reduce the risk of stunting or underweight. Antiretroviral therapy has been shown to improve LAZ and WAZ scores among HIV-infected children [28-38], but this is the first time that early extended exposure to nevirapine in infants has been shown to be associated with improved WLZ scores among HIV-exposed but uninfected infants. The extended dose of nevirapine was well tolerated by the infants in the SWEN arm of our study.

Interestingly, one study in India reported that pre-existing malnutrition impacted negatively on the nutritional response to ART [33]. Furthermore, a recent multicountry trial found that HIV-infected young children treated with a nevirapine-based ART had higher increases in Z scores for height and weight compared to those treated with a ritonavir-boosted lopinavir regimen [39]. We likely did not observe this finding in our HIV-infected cohort due to our small sample size of HIV-infected infants receiving SWEN. The mechanism for this presumed nevirapine effect however is unclear. Nevirapine has been studied in terms of antimicrobial properties but does not appear to have any specific activity against common bacteria impacting childhood illness [40], but it is not known if it has any effect on pediatric respiratory pathogens. To assess if other characteristics related to growth could explain our finding, we compared the duration of breastfeeding as well as the hospitalization rates between SWEN-exposed vs. SDNVP infants and found no significant differences. Interestingly, the FDA package insert for Nevirapine shows that the drug vehicle includes sorbitol and sucrose, but it is likely that this amount administered is too small to explain it’s effect on growth. While it could be a coincidental result, it is plausible that SWEN protected these infants from acquiring HIV infection, consequently protecting them against compromised nutritional status. Nevirapine may also have an effect on absorption of nutrients and on GI infections. Further understanding of the effect of nevirapine on growth is warranted. Although availability of cART in India has improved, the average age at which HIV-infected infants present for care in India is typically 6 years and many infants with perinatally acquired HIV infection remain undiagnosed and uninitiated on treatment [28].

We confirmed that importance of several of the maternal and infant factors that have been shown to be associated with poor growth in both HIV-unexposed and HIV-exposed infants in other settings. Maternal hemoglobin level remains a critical predictor of gestational weight gain [41]. It is routine practice to measure maternal hemoglobin levels during antenatal visits, and our data reinforce the need for women with lower hemoglobin levels to be identified during pregnancy should be more closely monitored and managed to prevent adverse outcomes for both maternal and infant health irrespective of the HIV status.

We found that low maternal education significantly increases the risk of stunting as have other studies [42-44]. Maternal education is not only a good measure of their knowledge of health-related issues, prenatal and postnatal infant care, infant feeding practices, and better sanitary habits, but it also impacts health-seeking behavior, income generating capacity, and ability to make autonomous decisions. Ascertaining the level of maternal education at first antenatal clinic contact in conjunction with measuring levels of malnutrition and anemia would be helpful to clinicians in providing adequate information and care to HIV-infected pregnant women and subsequently ensuring positive outcomes for infants. Similar to other studies, we also found that advanced disease status of the mother as measured by her CD4 cell count and HIV viral load at delivery also negatively impacts growth outcomes [20,45,46]. It is likely that mothers with low CD4 and/or higher viral load may be unable to provide adequate care for their infants because of the severity of their own illness [47-49]. Several of these factors are associated with maternal health and nutritional status both prior to and during delivery and therefore, emphasis on improving maternal health is critical to addressing adverse growth outcomes in infants, particularly if mother is HIV-infected. Infant factors such as low birth weight, preterm delivery and delivery mode other than normal vaginal delivery were all factors that significantly increased the risk of stunting, underweight, and wasting. Additionally, our results highlight the importance of breastfeeding among HIV-exposed infants.

As with other studies, our study has several strengths and limitations. A major strength of our study is the assessment of timing of infection which is likely to be more accurate due to multiple and frequent testing. While our study had excellent infant HIV diagnosis ascertainment, few children received ART as it was not available in the public sector at the time of our study. We however had prospective follow-up with multiple growth parameter assessments along with excellent retention, therefore our growth data from Indian infants are among the most robust. The limitation of our study was the lack of maternal BMI data, which is an important characteristic impacting infant growth. We also had limited information about weaning practices, especially between 6 and 9 month visits, when solid foods are introduced. Furthermore, our data come from a single site clinical trial where infants received enhanced care so some of our findings may not be generalizable to other settings; however since many children with HIV exposure are managed in urban public sector settings in India, we feel our data contribute relevant observations.

## Conclusions

Our study of growth patterns of infants born to HIV-infected mothers has revealed interesting and important findings not previously reported for such infants in India. Differences in anthropometric indices between infected and uninfected infants appear within 4–6 weeks of birth which is much earlier than 3 months to 1 year reported by previous studies. This emphasizes the need for monitoring their growth during the early months after birth. SWEN appeared to have a beneficial impact on growth especially among HIV-uninfected infants, and significantly reduced the risk of wasting. The high rates of maternal and infant malnutrition in India, and the low availability of early infant HIV testing suggests that early growth monitoring of all HIV-exposed infants coupled with nutritional advice to the parents are important low-cost strategies for improving health and survival outcomes of these infants.

## Abbreviations

HIV: Human immunodeficiency virus; SWEN: Six-week extended nevirapine; sdNVP: Single dose nevirapine; WAZ: Weight-for-Age standardized Z score; WLZ: Weight-for-Length standardized Z score; LAZ: Length-for-Age standardized Z score; SD: Standard Deviation; WHO: World Health Organization; PCR: Polymerase chain reaction; DNA: Deoxyribonucleic acid; RNA: Ribonucleic acid; CD4: Cluster of differentiation 4.

## Competing interests

The authors declare that they have no competing interests.

## Authors’ contributions

RB and AG conceived the study and designed the research; MR and UN participated in developing study instruments and standard operating procedures; AAK, MK, and JS conducted the research; MR, UN, and NG created and maintained databases, and performed statistical analysis; MR, AG, RB, NG, and AS helped to draft the manuscript. All authors read and approved the final manuscript.

## Pre-publication history

The pre-publication history for this paper can be accessed here:

http://www.biomedcentral.com/1471-2334/12/282/prepub

## Supplementary Material

Additional file 1 **Table S1. **Proportion Stunted, and Mean Length-for-Age Z Score by Infant HIV Status. **Table S2. **Proportion Underweight by HIV Status (WAZ). **Table S3.** Proportion Wasted by HIV Status (WLZ). **Table S4. **Mean Length-for-Age Z Scores by Infant HIV Status and SWEN. **Table S5. **Mean Weight-for-Age Z Scores by Infant HIV Status and SWEN. **Table S6. **Mean Weight-for-Length Z Scores by Infant HIV Status and SWEN. **Table S7. **Comparison of indicators of malnutrition among infants (Birth – 6 Months) in the general population between India and selected African countries. Click here for file
