# Supplementary material for: Growth patterns among HIV-exposed infants receiving nevirapine prophylaxis in Pune, India
Source: BMC Infect Dis. 2012 Oct 31;12:282. doi: 10.1186/1471-2334-12-282 (PMC3556061; doi:10.1186/1471-2334-12-282)
Supplement: Additional file 1 — Table S1. Proportion Stunted, and Mean Length-for-Age Z Score by Infant HIV Status. Table S2. Proportion Underweight by HIV Status (WAZ). Table S3. Proportion Wasted by HIV Status (WLZ). Table S4. Mean Length-for-Age Z Scores by Infant HIV Status and SWEN. Table S5. Mean Weight-for-Age Z Scores by Infant HIV Status and SWEN. Table S6. Mean Weight-for-Length Z Scores by Infant HIV Status and SWEN. Table S7. Comparison of indicators of malnutrition among infants (Birth – 6 Months) in the general population between India and selected African countries. [file 1471-2334-12-282-S1.doc]

**Growth Paper Supplemental Material**

**Table S1 -- Proportion Stunted, and Mean Length-for-Age Z Score by Infant HIV Status**

| **Visit** | **N** | **Total**  **N=737** | | **HIV-Uninfected**  **N=644** | | **HIV-Infected**  **N=93** | | **p Value*** |
| --- | --- | --- | --- | --- | --- | --- | --- | --- |
|  |  | **n (%)** | **Mean** | **n (%)** | **Mean** | **n (%)** | **Mean** |  |
| Birth | 728 | 339 (47) | -1.73 | 295 (46) | -1.72 | 44 (48) | -1.84 | 0.795 |
| 2 Weeks | 649 | 336 (59) | -2.17 | 336 (60) | -2.16 | 50 (58) | -2.26 | 0.786 |
| 4 Weeks | 643 | 363 (56) | -2.19 | 315 (56) | -2.15 | 48 (59) | -2.46 | 0.684 |
| 6 Weeks | 644 | 363 (56) | -2.18 | 308 (55) | -2.11 | 55 (65) | -2.60 | 0.071 |
| 10 Weeks | 635 | 305 (48) | -2.05 | 256 (46) | -1.99 | 49 (59) | -2.40 | **0.031** |
| 14 Weeks | 625 | 304 (49) | -1.97 | 255 (47) | -1.92 | 49 (61) | -2.36 | **0.016** |
| 6 Months | 602 | 309 (51) | -2.05 | 265 (50) | -2.03 | 44 (63) | -2.38 | **0.040** |
| 9 Months | 589 | 329 (56) | -2.18 | 284 (55) | -2.12 | 45 (63) | -2.65 | 0.173 |
| 12 Months | 582 | 340 (58) | -2.26 | 288 (56) | -2.17 | 52 (80) | -2.98 | **<0.0001** |

*****p-values are for comparison of proportions using χ^2^ test

**Table S2 -- Proportion Underweight by HIV Status (WAZ)**

| **Visit** | **N** | **Total**  **N=737** | | **HIV-Uninfected**  **N=644** | | **HIV-Infected**  **N=93** | | **p Value*** |
| --- | --- | --- | --- | --- | --- | --- | --- | --- |
|  |  | **n (%)** | **Mean** | **n (%)** | **Mean** | **n (%)** | **Mean** |  |
| Birth | 732 | 190 (26) | -1.49 | 165 (26) | -1.47 | 25 (27) | -1.63 | 0.828 |
| 2 Weeks | 649 | 251 (39) | -1.79 | 217 (39) | -1.76 | 34 (39) | -1.95 | 0.933 |
| 4 Weeks | 642 | 242 (38) | -1.77 | 208 (37) | -1.74 | 34 (43) | -1.99 | 0.295 |
| 6 Weeks | 639 | 219 (34) | -1.74 | 185 (33) | -1.71 | 34 (41) | -1.98 | 0.142 |
| 10 Weeks | 636 | 215 (34) | -1.67 | 175 (32) | -1.61 | 40 (48) | -2.01 | **0.003** |
| 14 Weeks | 625 | 206 (33) | -1.58 | 168 (31) | -1.52 | 38 (48) | -2.02 | **0.003** |
| 6 Months | 605 | 180 (30) | -1.52 | 149 (28) | -1.45 | 31 (43) | -2.04 | **0.009** |
| 9 Months | 592 | 178 (30) | -1.45 | 149 (29) | -1.40 | 29 (40) | -1.87 | **0.044** |
| 12 Months | 583 | 186 (32) | -1.51 | 153 (29) | -1.44 | 33 (52) | -2.08 | **<0.0001** |

*****p-values are for comparison of proportions using χ^2^ test

**Table S3 -- Proportion Wasted by HIV Status (WLZ)**

| **Visit** | **N** | **Total**  **N=737** | | **HIV-Uninfected**  **N=644** | | **HIV-Infected**  **N=93** | | **p Value*** |
| --- | --- | --- | --- | --- | --- | --- | --- | --- |
|  |  | **n (%)** | **Mean** | **n (%)** | **Mean** | **n (%)** | **Mean** |  |
| Birth | 609 | 62 (10) | -0.24 | 57 (11) | -0.25 | 5 (7) | -0.15 | 0.299 |
| 2 Weeks | 626 | 59 (9) | -0.40 | 48 (9) | -0.38 | 11 (13) | -0.56 | 0.200 |
| 4 Weeks | 631 | 46 (7) | -0.06 | 41 (7) | -0.05 | 5 (7) | 0.13 | 0.825 |
| 6 Weeks | 636 | 36 (6) | 0.31 | 32 (6) | 0.29 | 4 (5) | 0.48 | 0.763 |
| 10 Weeks | 631 | 24 (4) | 0.26 | 19 (3) | 0.28 | 5 (6) | 0.25 | 0.256 |
| 14 Weeks | 625 | 37 (6) | 0.09 | 29 (5) | 0.11 | 8 (10) | -0.03 | 0.098 |
| 6 Months | 602 | 37 (6) | -0.10 | 28 (5) | -0.06 | 9 (13) | -0.37 | **0.017** |
| 9 Months | 590 | 29 (5) | -0.19 | 25 (5) | -0.16 | 4 (6) | -0.35 | 0.789 |
| 12 Months | 581 | 35 (6) | -0.41 | 32 (6) | -0.40 | 3 (5) | -0.49 | 0.656 |

*****p-values are for comparison of proportions using χ^2^ test

**Table S4 -- Mean Length-for-Age Z Scores by Infant HIV Status and SWEN**

| **Visit** | **HIV-Infected** | | | | **HIV-Uninfected** | | | |
| --- | --- | --- | --- | --- | --- | --- | --- | --- |
|  | **N** | **SWEN** | **Single Dose** | **p Value** | **N** | **SWEN** | **Single Dose** | **p Value** |
| Birth | 92 | -1.64 | -2.00 | 0.103 | 636 | -1.77 | -1.66 | 0.246 |
| 2 Weeks | 86 | -2.02 | -2.45 | 0.086 | 563 | -2.21 | -2.11 | 0.261 |
| 4 Weeks | 82 | -2.21 | -2.66 | 0.125 | 561 | -2.17 | -2.13 | 0.711 |
| 6 Weeks | 84 | -2.35 | -2.78 | 0.145 | 560 | -2.12 | -2.10 | 0.837 |
| 10 Weeks | 83 | -2.23 | -2.52 | 0.354 | 552 | -2.04 | -1.94 | 0.344 |
| 14 Weeks | 80 | -1.89 | -2.71 | **0.007** | 545 | -1.95 | -1.88 | 0.513 |
| 6 Months | 70 | -2.17 | -2.55 | 0.271 | 532 | -2.02 | -2.03 | 0.943 |
| 9 Months | 71 | -2.40 | -2.86 | 0.169 | 518 | -2.10 | -2.13 | 0.719 |
| 12 Months | 65 | -2.69 | -3.23 | 0.099 | 517 | -2.21 | -2.13 | 0.489 |

**Table S5 -- Mean Weight-for-Age Z Scores by Infant HIV Status and** SWEN

| **Visit** | **HIV-Infected** | | | | **HIV-Uninfected** | | | |
| --- | --- | --- | --- | --- | --- | --- | --- | --- |
|  | **N** | **SWEN** | **Single Dose** | **p Value** | **N** | **SWEN** | **Single Dose** | **p Value** |
| Birth | 92 | -1.44 | -1.77 | 0.130 | 639 | -1.50 | -1.44 | 0.490 |
| 2 Weeks | 86 | -1.76 | -2.10 | 0.202 | 562 | -1.77 | -1.75 | 0.828 |
| 4 Weeks | 78 | -1.78 | -2.15 | 0.191 | 563 | -1.73 | -1.75 | 0.831 |
| 6 Weeks | 81 | -1.71 | -2.19 | 0.093 | 557 | -1.69 | -1.72 | 0.732 |
| 10 Weeks | 82 | -1.77 | -2.20 | 0.125 | 553 | -1.56 | -1.64 | 0.600 |
| 14 Weeks | 79 | -1.69 | -2.26 | 0.074 | 545 | -1.45 | -1.59 | 0.146 |
| 6 Months | 71 | -1.95 | -2.11 | 0.659 | 533 | -1.37 | -1.54 | 0.068 |
| 9 Months | 71 | -1.66 | -2.03 | 0.225 | 520 | -1.30 | -1.50 | **0.036** |
| 12 Months | 64 | -2.03 | -2.12 | 0.771 | 519 | -1.43 | -1.46 | 0.755 |

**Table S6 -- Mean Weight-for-Length Z Scores by Infant HIV Status and SWEN**

| **Visit** | **HIV-Infected** | | | | **HIV-Uninfected** | | | |
| --- | --- | --- | --- | --- | --- | --- | --- | --- |
|  | **N** | **SWEN** | **Single Dose** | **p Value** | **N** | **SWEN** | **Single Dose** | **p Value** |
| Birth | 74 | -0.16 | -0.15 | 0.981 | 535 | -0.29 | -0.22 | 0.496 |
| 2 Weeks | 83 | -0.63 | -0.51 | 0.648 | 543 | -0.34 | -0.42 | 0.472 |
| 4 Weeks | 75 | 0.17 | 0.09 | 0.804 | 556 | 0.10 | -0.01 | 0.329 |
| 6 Weeks | 81 | 0.64 | 0.36 | 0.379 | 555 | 0.32 | 0.26 | 0.589 |
| 10 Weeks | 83 | 0.37 | 0.16 | 0.449 | 548 | 0.40 | 0.15 | **0.024** |
| 14 Weeks | 80 | -0.15 | 0.05 | 0.520 | 545 | 0.25 | -0.02 | **0.012** |
| 6 Months | 72 | -0.45 | -0.31 | 0.709 | 530 | 0.07 | -0.20 | **0.010** |
| 9 Months | 72 | -0.26 | -0.41 | 0.570 | 518 | -0.03 | -0.30 | **0.005** |
| 12 Months | 63 | -0.50 | -0.48 | 0.921 | 518 | -0.37 | -0.43 | 0.525 |

**Table S7 - Comparison of indicators of malnutrition among infants (Birth – 6 Months) in the general population between India and selected African countries**

| Country | Year | % Underweight  WAZ <-2.0 SD | % Stunted  HAZ <-2.0 SD | % Wasting)  WHZ <-2.0 SD |
| --- | --- | --- | --- | --- |
| India | 2005-06 | 32.2 | 22.6 | 30.5 |
| South Africa  - KwaZulu-Natal  - Eastern Cape | 2003  2003 | 5.1  4.1 | 8.8  16.8 | 5.9  3.1 |
| Kenya | 2003 | 8.1 | 15.8 | 8.5 |
| Uganda | 2006 | 11.1 | 17.9 | 8.8 |
| Ethiopia | 2005 | 13.6 | 19.2 | 15.8 |
| Rwanda | 2005 | 9.8 | 18.5 | 7.2 |
| Zimbabwe | 2005-06 | 9.7 | 18.8 | 11.1 |

Source: WHO Global Database on Child Growth and Malnutrition
